# Discharge Plan to Promote Patient Safety and Shared Decision Making by a Multidisciplinary Team of Healthcare Professionals in a Respiratory Unit

**DOI:** 10.3390/nursrep11030056

**Published:** 2021-07-30

**Authors:** Daniel A. Nnate, David Barber, Ukachukwu O. Abaraogu

**Affiliations:** 1 Department of Nursing and Community Health, School of Health and Life Sciences, Glasgow Caledonian University, Glasgow G4 0BA, UK; David.Barber@gcu.ac.uk; 2 Department of Physiotherapy and Paramedicine, School of Health and Life Sciences, Glasgow Caledonian University, Glasgow G4 0BA, UK; Ukachukwu.Abaraogu@gcu.ac.uk

**Keywords:** chronic obstructive, healthcare leadership, patient discharge, personalized care, interdisciplinary health team, patient safety, pulmonary disease, pulmonary medicine

## Abstract

Patients with chronic obstructive pulmonary disease (COPD) often require frequent hospitalization due to worsening symptoms. Preventing prolonged hospital stays and readmission becomes a challenge for healthcare professionals treating patients with COPD. Although the integration of health and social care supports greater collaboration and enhanced patient care, organizational structure and poor leadership may hinder the implementation of patient-oriented goals. This paper presents a case of a 64-year-old chronic smoker with severe COPD who was to be discharged on long-term oxygen therapy (LTOT). It also highlights the healthcare decisions made to ensure the patient’s safety at home and further provides a long-lasting solution to the existing medical and social needs. The goal was accomplished through a discharge plan that reflects multidisciplinary working, efficient leadership, and change management using Havelock’s theory. While COPD is characterized by frequent exacerbation and hospital readmission, it was emphasized that most failed discharges could be attributed to bureaucratic organizational workflow which might not be in the patient’s best interest. It was further demonstrated that healthcare professionals are likely to miss the window of opportunity to apply innovative and long-lasting solutions to the patient’s health condition in an attempt to remedy the immediate symptoms of COPD.

## 1. Introduction

Over the years, there has been a continuous restructuring of primary health care from policies to technology to accommodate the growing population size and the complex health needs of society [1]. The main objective is to promote multi-disciplinary team (MDT) working among public, private and third sectors to improve practice, provide stronger support for collaborative healthcare decisions and build sustainable healthcare policies. It may be considered that a functional health system encompasses diverse professionals coming together for the benefit of the patient [2,3]. This includes but is not limited to a team of physicians, occupational therapists, nurses, laboratory technicians and physiotherapists. Inter-professional collaboration in primary care is a partnership between different health professionals, blending complementary competencies and skills, and making possible the best use of resources through shared decision making to mitigate health and social issues [4]. The World Health Organization places much emphasis on interprofessional collaboration in healthcare delivery to ensure patient safety, and further regards such practice as a framework for universal health coverage [5,6]. While integrating health and social care supports greater collaboration and enhanced patient care, healthcare decisions to promote the safety and self-determination of patients becomes a challenge when dealing with complex discharge to avoid a prolonged hospital stay and readmission [7,8]. Given the potentially huge contrast between upholding patient’s autonomy and following clinical guidelines, or maintaining organizational values, health practitioners may face certain challenges in decision-making to support the best interest of patients.

Chronic obstructive pulmonary disease (COPD) is an inflammatory lung disease that causes obstructed airflow from the lungs. It is often caused by long-term exposure to irritating gases or particulate matter, most often from cigarette smoke. The progressive nature of the disease is characterized by increased breathlessness, persistent cough, sputum production, wheezing and fatigue. End-stage COPD is not reversible. However, lifestyle changes and treatments can help slow the progress of the disease, prevent further complications, and improve the quality of life for patients with COPD. This further calls for innovative, acceptable, and evidence-based interventions that could reduce morbidity and mortality associated with COPD patients. Wouters et al. [9] proposed that management of patients with COPD should take an integrated personalized approach with input from various healthcare professionals and in partnership with the patient. This approach puts the patient at the center of healthcare decisions and aims to promote patient autonomy, self-management of symptoms and improved psychosocial needs and quality of life.

Pulmonary rehabilitation aims to minimize the physical and emotional impacts of a chronic lung condition on quality of life through exercise training, psychosocial intervention, and patient education [10]. Initiating pulmonary rehabilitation within 90 days was reported to be associated with lower risk of death, which is even more effective when commenced very early, within 30 days of discharge [11]. It is one of the most effective treatments for COPD and other chronic pulmonary diseases such as asthma, interstitial lung disease, and lung cancer [12,13]. Despite available evidence on the benefits of pulmonary rehabilitation in clinical practice, and recommendations for its use in international guidelines [14], it is still underutilized. Only 2–4% of patients hospitalized with COPD exacerbation are being referred for pulmonary rehabilitation in the US [11,15] and less than 17% of patients referred after admission for exacerbation of COPD commence pulmonary rehabilitation within 30 days in the UK [16,17]. In their study, Early et al. [17] reported that the major barrier to referral and initiation of pulmonary rehabilitation in patients were varied, with weak referral pathways and lack of enthusiasm from patients being the most common.

The personalized medicine approach for managing COPD relies on a collaborative effort between the patient and healthcare professionals which is based on trust and mutual understanding in order to move from the current status quo of relieving the immediate symptoms upon hospitalization to innovative nonpharmacologic COPD treatments [9]. As patients with frequent exacerbation often require hospitalization due to worsening symptoms [18], the collaboration between acute and community health services is apparent in the need to establish patient-centered goals and follow-up after discharge. This paper aims to explore multidisciplinary health care decision making in providing safe and person-centered care through pulmonary rehabilitation upon discharge that agrees with organizational values from a practice scenario. It entails a brief description of the situation, identifying the stakeholders involved, patient referral and analysis of the situation to reflect multidisciplinary working, and efficient leadership and change management using Havelock’s theory of change [19]. This will be followed by a critique of the change process to identify any merits and its perceived shortcomings.

## 2. Case Report

The practice situation presented was encountered in a general respiratory ward. A pseudonym was adopted instead of real names to preserve the patient’s anonymity. The individual who will be referred herein as Mary is a 64-year-old chronic smoker who lives alone with support from her daughter. She was admitted with increased shortness of breath, confusion, and hypoxia (oxygen saturation [SPO2] at 71%). Post-bronchodilator spirometry indicated a forced expiratory volume in 1 s (FEV1) to forced vital capacity (FVC) ratio below 0.70, with bronchodilator responsiveness below 30–49% FEV1. This signifies an airflow limitation peculiar to severe chronic obstructive pulmonary disease (COPD) [20,21]. Mary was then placed on oxygen therapy but seems to be non-compliant as she was caught smoking in the hospital ward. She had previously enrolled in smoking cessation programs but failed after a couple of attempts. After one week of hospitalization, she now wishes to go home. With Mary’s SPO2 below 90% on air, a partial pressure of oxygen (PaO2) of 7.5 kPa, and a normal partial pressure of carbon dioxide (PaCO2), she was a candidate for long-term oxygen therapy (LTOT) [22]. The MDT faced making a discharge plan to support Mary’s choice and put in place safety measures to prevent her from being readmitted, and moreover, prevent a fire hazard if she was to be discharged with LTOT.

The intricacies of this case scenario were pertinent to delivering safe and effective care while respecting Mary’s right to self-determination. Therefore, healthcare workers were confronted with the need to consider the “patient’s best interests” and “best medical interests” in their decision-making. In this context, the MDT accountable for Mary’s care consisted of a respiratory consultant, smoking cessation psychologist, respiratory specialist nurse, and a physiotherapist. Notwithstanding, Mary and her daughter were also involved in the decision-making process. Regarding the practice scenario, in the next section, we will further evaluate the issues presented and their implications on leadership, inter-professional working, organizational values and professional values for each member of the MDT involved in Mary’s care.

## 3. Discussion

### 3.1. Analysis of Organizational Structure and Clinical Guidelines

**Figure 1:**
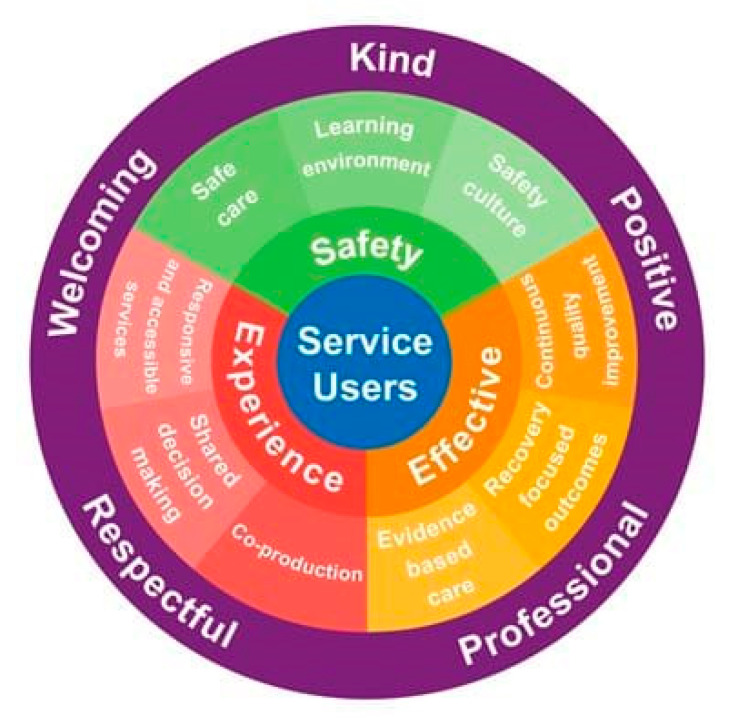
Values of the NHS Constitution [31].

In healthcare, guidelines are established based on evidence to ensure consistency in health care delivery and continuity of care [23,24]. Adhering to guidelines and recommendations does not only guarantee good practice but is also embedded in the code of conduct of healthcare professionals [25,26,27]. Furthermore, organizational values are also founded based on guidelines and standards of behavior that are shared by a team of health professionals. For instance, healthcare providers such as the National Health Service (NHS) in the UK gain public trust and ensure that patients receive the best possible care by maintaining core values which are embedded in its constitution [28,29] (see Figure 1). These values are demonstrated through teamwork, quality of care delivery, openness, compassion, dignity, honesty and responsibility, and respect for patients.

Organizational structure has been perceived as a hindrance to implementing positive change with regard to healthcare decisions. The levels of authority and staff responsibilities creates less opportunities for innovation due to increased bureaucracy. Therefore, most decisions are often inclined towards maintaining organizational structure and workflow. However, healthcare decisions might not be in the interest of the patient’s wellbeing. While the British Thoracic Society (BTS) guidelines on oxygen therapy are based on the best evidence, it has been emphasized that guidelines are not substitutes for clinical judgement [22,30].

### 3.2. Discharge Plan

A change management approach was utilized to plan Mary’s discharge to the community. This involves leadership support to oversee the activities of the multidisciplinary team while allowing team members to express their ideas and any concerns they might have regarding Mary’s safety at home. Led by the respiratory consultant, the MDT convened with each member highlighting their concern. To discharge Mary on LTOT, the nurse practitioner advised that strict measures must be taken to break her tobacco dependence. It was also certain that Mary’s action in the ward puts her at risk of setting her home ablaze. The physiotherapist further proposed that despite Mary’s recent exacerbation, she may still benefit from pulmonary rehabilitation. The consultant also emphasized the need to have supplemental oxygen in place for potentially avoidable deterioration from respiratory failure. To successfully help Mary through quitting her smoking habit, the psychologist suggested that helping her to maintain a healthy behavior change was necessary [32]. While Mary’s situation may require the clinical team to act in opposition to the BTS guidelines for home oxygen therapy in adults, dealing with the concern raised required exceptional team leadership. Therefore, bearing in mind the effort needed to implement change, it may be considered that leading and managing change requires good leaders who can successfully influence the change process.

### 3.3. Leadership and Change Management

Change can be effectively implemented with excellent leadership skills that can influence the activities of a group towards a desired goal [33,34]. Change is often triggered by the need to deal with the complexities of organizational structures, and guidelines that emanate from the application of procedures and skills that may be inappropriate to solve certain problems [34]. Hospitalization during exacerbations is perceived as a major contributing factor to increased morbidity and mortality in COPD patients, as oftentimes healthcare professionals rely on conventional bronchodilators as opposed to pulmonary rehabilitation due to perceived uncertainty and unpredictability. Although the MDT projected a deeper level of change, the clinician saw the need to first inspire team members to reach their maximum potential and be accountable for their actions in their quest to meet Mary’s need in a transformational leadership style [35,36]. The transformational leadership approach has been recommended as an essential means of leading organizational change [37]. It requires the leader to work together with each team member based on individual knowledge and skill to inspire confidence and devotion to the set goal. Furthermore, the ability to manage uncertainties that may ensue and unpredictability in Mary’s behavior will enrich future leader-follower relationships.

To successfully manage the required change process, it is necessary to build trust, loyalty, and mutual benefit within the team. This reciprocity between the team leader and members of the MDT is well described in the social exchange theory which states that relationships are built on the ability of each party to maximize what they stand to gain [38]. Consequently, when other health professionals feel their efforts outweigh the benefit, the proposed task may not be performed. Thus, there is a need to maintain clarity and mutual benefit in order to sustain a working relationship between transformational leaders and their followers. In their survey, Steinmann et al. [39] demonstrated that transformational leaders are able to influence the extent to which followers regard organizational goals as important and attainable. To instill positive attitudes and proactive behavior on followers, Hussain et al. [37] further recommends that leaders must be flexible in their approach but stay focused on the goal. The transformational change in practice was further made feasible using an ethical leadership approach. Ethical leadership in healthcare entails making decisions with maximum effect from a list of available choices that thrive for fairness, show respect for beliefs, rights and values of patients and staff alike [35,40]. According to Northouse [35], respect for people is a unique characteristic of transformational and ethical leaders. This is evident in the leadership style which was directed at strengthening organizational values, personal growth, and instilling trust among the MDT members.

Lewin [41] described the change process as a state of unfreezing the current situation, attain the desired change and then refreeze for a stable result. Havelock [19], however, suggested that the linear process may not apply to real-life situations, and further recommended the need to build trust with the subject and put specific plans in place to monitor progress once the change is implemented. Havelock’s change theory has been recommended for overcoming barriers and empowering team members when making complex decisions in healthcare [42,43]. This model readily supports the application of the World Health Organization’s health service planning and policy implementation toolkit [44]; the health behavior change competency (HBCC) framework, a Public Health Scotland recommended approach to delivering interventions for health behaviour change [32] (see Table 1). The change in practice that was required for Mary’s successful discharge will be further analyzed using Havelock’s six-phase model: (a) building relationship, (ii) diagnosing the problem, (iii) acquiring relevant resources, (iv) choosing an appropriate solution, (v) gaining acceptance, and (vi) stabilization and self-renewal [19].

According to Havelock [19], establishing a patient–care relationship is required to see the proposed transformation from the patient’s viewpoint. This is considered the first step to implementing change. Therefore, by adopting a person-centered approach to meeting Mary’s health needs, the nurse practitioner took up clinical responsibilities in the community while the physiotherapist was to deliver exercise training to fit in with her personal goals and progress. A further assessment of Mary’s health literacy shows that she now understands the nature of her health condition and the implications where appropriate measures are not taken. She also demonstrated her ability to identify unhealthy behaviors that could trigger an exacerbation of her condition and understood the benefits of pulmonary rehabilitation and components of the treatment plan. While the patient was deemed to have the capacity to make informed decisions and does not feel under pressure to comply, she was also reminded of her right to opt out at any time.

The second stage which has to do with diagnosing the problem was steered by the smoking cessation psychologist. Barriers that prevented Mary from quitting her smoking habits were explored, and she also admits getting tobacco supplies from her daughter. Further assessment revealed that Mary was a suitable candidate for behavioral change modification. Dixon and Johnston [32] assert that the contemplation step in diagnosing the problem often drives the change process. Mary’s commitment to change was followed up with her enrollment into the smoking cessation program. Goals were also set to gradually cut down the number of cigarettes she smoked per day. Relaxation therapy was also incorporated into the pulmonary rehabilitation program to reduce anxiety, triggered by increased breathlessness. Selebi et al. [45] advocated for the inclusion of psychological interventions along with other therapies, such as breathing exercises and meditation to reduce anxiety.

The third stage was led by the respiratory consultant with support from the nurse specialist. It entailed getting resources to support the treatment plan, such as referring to the relevant evidence for home oxygen use in adults with COPD. Although the BTS advises that patients with SPO2 ≤ 92% should be assessed for LTOT, further recommendation suggests that an exacerbation of COPD may cause temporary worsening of hypoxemia even at the point of hospital discharge [22]. However, it was emphasized that hypoxemia may improve within two months with optimal treatment of the cause of exacerbation [22,46].

In the fourth step, an appropriate pathway for the proposed change was selected. Following clinical recommendations, Mary’s medication was to be constantly reviewed for maximum therapeutic effect and the nurse practitioner was to oversee her adherence and concordance to medications. It was then agreed that Mary would be discharged without LTOT. In addition, supplemental oxygen was kept on standby to avoid further deterioration and exercise-induced desaturation. However, recent evidence suggested that that the non-availability of supplemental oxygen should not be a barrier to exercise training [47]. Mary was also trained in measuring her FEV1 value at home. The value of FEV1 over time can show a rapid decline in lung function in COPD patients [48,49]. Any sign of deterioration was passed on to the medical team.

The World Health Organization [44] advocates that any resistance to the proposed change must be identified for a sustainable impact. Hence, the fifth step was directed towards establishing and accepting the preferred solution. The feasibility of maintaining the change was further assessed using a force field analysis (see Table 2). While smoking is recognized as the major cause of fire hazards in patients on LTOT, support systems such as smoking cessation programs have been recommended to help patients on LTOT to pull through [22,50]. As progress is often self-reported, it may not accurately reflect the perceived change. In order to prevent a relapse in Mary’s progress, much emphasis was paid on the need for Mary’s daughter to step in as an advocate of the smoking cessation and pulmonary rehabilitation program.

The final stage of Havelock’s change model is maintenance and separation. This involved continuous monitoring and evaluation of Mary’s care and the progress of each team member. Schyns and Schilling [51] advised that negative attitudes of team members towards the set goal often emanates from a lack of faith in the subject or entire process. The clinician showed support for the MDT to feel safe in expressing their concerns in order to address any conflicting values. This portrayed certain traits of a value-based leader who seeks openness, courage, and commitment to quality of care as described by Stanley [52].

### 3.4. Force Field Analysis

The current intervention was anticipated to last for about 2–3 months, which is intended to provide a long-lasting solution for Mary. Evidence suggests that early symptoms of COPD may be partially reversible with appropriate treatment [22,45]. Guyatt et al. [46] previously reported a 36–51% symptom stability between 2 months and 1 year in patients without LTOT compared to patients immediately started on LTOT after an exacerbation. Hence, it was evident that goals which are meant to be achieved over a long period require team members to be compliant and fully committed. Table 2 highlights the drivers for change required to promote positive change, and restraining forces that may likely hinder the team’s attitude towards change.

Udod and Wagner [42] further suggested that challenges often encountered in healthcare decision-making emanates from the complexities of dealing with human lives due to conflicting values. While organizational and ethical values promote the delivery of holistic person-centered care and respect for human dignity, these values may likely hinder innovation in healthcare decision making. Hardinge et al. [22] also reported that even where LTOT is not required, patients may feel distressed when LTOT is withdrawn, and healthcare workers may find this challenging. This is likely to lead to extended use of oxygen therapy. Although good leadership played a major role in Mary’s discharge, having a consistent goal and providing an avenue for other healthcare professionals to share their thoughts helps to keep the team motivated without anyone feeling left out.

## 4. Conclusions and Recommendations

This study set out to evaluate a complex discharge plan to support the safety and self-determination of a patient with an exacerbation of COPD. From the practice scenario, it can be argued that MDT working in acute and community health services has its benefits and may be the preferred approach to minimize long hospital stays and readmission rates. However, delivering personalized care to enhance the patient’s quality of life requires a leadership style that considers organizational values while paving way for personal development within the team. Although the approach adopted by each team member was professionally acceptable to meet the patient’s needs, tension may arise amongst the MDT because of a lack of enthusiasm in the patient or team members. The transformational and ethical leadership style employed to lead this change was made to build trust and support the team members to feel safe to express their concerns. This helped to eliminate any resistance that could hinder the change progress and thus provide a long-lasting solution for the patient.

Mary’s successful discharge presents an instance where addressing a patient’s social needs within healthcare delivery becomes significant to achieving a long-term solution with a better quality of life. In an attempt to remedy the immediate symptoms of COPD, healthcare professionals often miss the window of opportunity to apply innovative and long-lasting solutions to the patient’s health condition. This further creates a cycle of re-hospitalization, worsening symptoms and poor quality of life for the patient. Most failed discharges have been attributed to organizational workflow that seems to be task oriented. Although it has been emphasized that good leadership is pertinent to achieving organizational change, the ability to envision the required transformation from a holistic standpoint is essential for effective change management in healthcare.

## Figures and Tables

**Table 1:** Change models for healthcare decision-making.

Havelock’s Six-Stage Model [19]	Health Behavior Change Competency (HBCC) Framework [32]	Health Service Planning and Policy-Making Toolkit for Nurses and Midwives [44]
Building relationship	Pre-contemplation	Defining the problem
Diagnose problem	Contemplation	Stakeholder analysis and networks
Acquire resources	Preparation	Assessing contextual issues
Choose solution	Action	Policy-development process
Gain acceptance	Maintenance	Communication and change management
Stabilization and self-renewal	Termination	Monitoring and evaluation

**Table 2:** Force field analysis.

Drivers for Change	Restraining Forces
Good leadership Personal values Role modeling Self-awareness Supportive staff Good collaboration Strong communication Ethical behavior Speaking up Showing respect for patients	Lack of motivation Conflicting opinions Cost effectiveness Inconsistency Adherence Environments that do not support change Feeling of not fitting in Policies Poor family support

## Data Availability

No new data were created or analyzed in this study. Data sharing is not applicable to this article.
